# Photosynthesis of the *Cyanidioschyzon merolae* cells in blue, red, and white light

**DOI:** 10.1007/s11120-020-00796-x

**Published:** 2020-11-24

**Authors:** Eugeniusz Parys, Tomasz Krupnik, Ilona Kułak, Kinga Kania, Elżbieta Romanowska

**Affiliations:** Department of Molecular Plant Physiology, Faculty of Biology, University of Warsaw, Miecznikowa 1, 02-096 Warsaw, Poland

**Keywords:** Photosynthesis and respiration, Light quality, CO_2_ and O_2_ exchange, Chlorophyll *a*, Zeaxanthin and β-carotene, Red alga, *Cyanidioschyzon merolae*

## Abstract

Photosynthesis and respiration rates, pigment contents, CO_2_ compensation point, and carbonic anhydrase activity in *Cyanidioschizon merolae* cultivated in blue, red, and white light were measured. At the same light quality as during the growth, the photosynthesis of cells in blue light was significantly lowered, while under red light only slightly decreased as compared with white control. In white light, the quality of light during growth had no effect on the rate of photosynthesis at low O_2_ and high CO_2_ concentration, whereas their atmospheric level caused only slight decrease. Blue light reduced markedly photosynthesis rate of cells grown in white and red light, whereas the effect of red light was not so great. Only cells grown in the blue light showed increased respiration rate following the period of both the darkness and illumination. Cells grown in red light had the greatest amount of chlorophyll a, zeaxanthin, and β-carotene, while those in blue light had more phycocyanin. The dependence on O_2_ concentration of the CO_2_ compensation point and the rate of photosynthesis indicate that this alga possessed photorespiration. Differences in the rate of photosynthesis at different light qualities are discussed in relation to the content of pigments and transferred light energy together with the possible influence of related processes. Our data showed that blue and red light regulate photosynthesis in *C. merolae* for adjusting its metabolism to unfavorable for photosynthesis light conditions.

## Introduction

### Taxonomic position, classification, and physiology of *Cyanidioschyzon merolae*

The *Cyanidioschyzon merolae* is a member of *Cyanidiales*, and order of red algae, it is a small (1.5 × 3.5 μm in size) thermo-acidophilic unicellular alga isolated from an Italian volcanic hot springs (Merola et al. 1981). It grows best at a pH of 1.5 and a temperature of 45 °C (Albertano et al. 2000). *Cyanidiales* are only phototrophic organisms and eucaryotes found in acidic hot water. The cell has an extremely simple structure, with one nucleus, one mitochondrion, and one chloroplast (Kuroiwa et al. 1994). The full nucleotide sequences of these three genomes were determined. There is coordinated gene expression between the chloroplast and the mitochondrion, and the expressions of genes of both organelles are regulated both by light and by the cell cycle (Kanesaki et al. 2012). Phylogenetic analyses of photosynthetic genes suggest that *C. merolae* is one of the most primitive photosynthetic eukaryotes and that it diverged just after the monophyletic origin of plastids (Nozaki et al. 2003). Phylogenetic studies based on sequence of plastid genes have shown that strains of *C. merolae* can be found in distantly located hot springs, regardless of their geographic origins (Ciniglia et al. 2004). Thus, they probably migrated between separated springs.

*Cyanidioschyzon merolae* does not appear red but blue-green in color because besides chlorophyll *a* (Chl *a*) and the carotenoid zeaxanthin (Zea) and β-carotene (β-Car) its chloroplast possesses phycobiliprotein phycocyanin (PC) and allophycocyanin (APC) in phycobilisomes (PBSs), which together absorb the broad red and blue wavelengths of the white light (Cunningham et al. 2007; Su et al. 2010). Since phycoerythrin (PE), a red pigment that was absorbed in the blue-green region, is absent, *C. merolae* is not capable of chromatic adaptation (Talarico and Maranzana 2000). In addition, it lacks photoreceptors such as phytochrome and phototropin, while genes encoding cryptochrome-related proteins (cryptochrome and photolyase) are present and expressed (Kanesaki et al. 2012; Asimgil and Kavakli 2012). Furthermore, the alga contains ribulose 1,5-bisphosphate carboxylase/oxygenase (Rubisco) and other enzymes, and metabolites related to essential metabolic processes (Fujita et al. 2008; Moriyama et al. 2014; Rademacher et al. 2016). Like the other tiny (about twice smaller) phototrophic organism, *Ostreococcus tauri*, which belongs to a group of green algae called prasinophytes (Courties et al. 1994; Archibald 2006), *C. merolae* does not have a characteristic cell wall. This may have relevance to its high cell surface-to-cell volume ratio (thus the abundance of diffusing CO_2_) and lack of typical vacuoles. Instead, *C. merolae* possess tiny vacuoles (500 nm in diameter, about five per interphase cell), identified as polyphosphate-containing vacuoles (Yagisawa et al. 2007, 2009), which occupy less than 10% of the cell volume. Therefore, the cells of this alga do not break even if they are immersed in distilled water.

Similar to other red algal species, *C. merolae* has only Chl *a* in both photosystems, PSI and PSII. The majority of Chl *a* is localized in PSI, forming a light-harvesting antennal complex associated with the core of the photosystem. Chlorophyll *a* as a minor component is also localized in the core of PSII and its low amount is compensated by the abundance of PBSs, the major protein light-harvesting complexes in the photosystem (Enami et al. 2010; Su et al. 2010). When photosynthesis proceeds, the electrons are transported from H_2_O to CO_2_, which is manifested by chloroplasts as CO_2_ uptake and O_2_ evolution in equivalent amounts, but these two reactions are unnecessarily performed by whole cells if, for example, the photo-/respiratory processes are highly active. The latter events are of no importance in the stoichiometry of CO_2_/O_2_ exchange in *C. merolae* because the ratio between the photosynthetic activity carried out by the large chloroplast occupying more than half of the cell volume (Suzuki et al. 1994) and respiration rate is incomparable (as reported here).

It is worth to note that Zenvirth et al. (1985) were probably the first to examine the photosynthetic activity of *C. merolae*. They noticed that the rate of O_2_ evolution from illuminated cells decreased with the increase in pH from 1.5 to 5.5. Interestingly, at pH 3.5, the rate of O_2_ evolution (~ 130 µmol O_2_ mg^−1^ Chl) was almost the same as we noted for the mesophyll protoplasts of *Pisum sativum* at pH 7.6, which showed, besides, 12-fold lower rate of dark respiration following photosynthesis compared with that of previous photosynthesis (Parys et al. 1998). But others noticed markedly low photosynthesis as well as a low photosynthesis to respiration ratio for *C. merolae* (Rademacher et al. 2016; Nikolova et al. 2017). This suggests that the photosynthetic activity of *C. merolae* cells may be susceptible to factor(s) that are usually not considered as significant or cells exhibited diminished physiological parameters.

Light quality and photosynthesis.

Data on the effect of light quality on photosynthetic activity of *C. merolae* cells are not available as yet. As it was established, only the influence of different colors of light (blue, green, yellow, and red with a single peak at 460, 515, 590, and 666 nm, respectively) on energy transfer from PBS to PSII and PSI, and on the relative amount of pigments, has been investigated (Ueno et al. 2015). The energy of the photons absorbed by PC and APC (*λ*_max_ = 620 and 652 nm, respectively) (Adir 2005), Chl *a* (*λ*_max_ = 431 and 663 nm) (Cunningham et al. 2007), and possibly β-Car (*λ*_max_ = 482 nm) (Goedheer 1969; Cunningham et al. 2007) is funneled to the reaction center (RC) of the photosystems where Chl *a* uses only red photons/excitons to drive water-splitting and ferrodoxin-reducing photochemistry (Björn et al. 2009; Mirkovic et al. 2017). From the higher excited state that resulted from the absorption of blue photons (425–490 nm), Chl *a* drops down to the first singlet state, at which there is energy available only for photosynthesis. Then, the same amount of the absorbed blue photons results in the same excited state as red photons absorbed by Chl *a* and subsequently, in comparable rates of electron transport, O_2_ evolution, or CO_2_ uptake. Results in this field are available from relevant studies on red algal species, however, other than *C. merolae*, but these are not always conclusive. Figueroa et al. (1995) showed that the thallus of red macroalga *Porphyra umbilicalis* cultured in blue (*λ*_max_ = 465 nm) and red light (*λ*_max_ = 650 nm) did not show any difference in the rate of photosynthetic O_2_ evolution in white light (400–700 nm), expressed in terms of Chl *a* or total pigments (Chl *a*, PC, PE). While expressed in dry weight or area units, the rate was found to be several-fold higher in the thallus grown in blue light compared with that grown in red light, similar to the content of pigments expressed per unit dry weight. In another species, *Porphyra leucosticta*, the rate of electron transfer (following white light pulses) in the thallus cultivated in blue light (*λ*_max_ = 465 nm) was much lower than that cultivated in red light (*λ*_max_ = 657 nm), though both samples had no great differences in the pigments (Chl *a* and PC) expressed per unit dry weight (Korbee et al. 2005). These results coincide with the data showing that photosynthetic activity (expressed per unit dry weight) in the above alga that was determined at the same light quality as during growth was lower by more than twofold in blue (400–500 nm) than in red (600–700 nm) light; nonetheless, the sum of photosynthesis in blue and red light resulted in almost the same rate as in white (400–700 nm) light (Aguilera et al. 2000). This agrees with the data from the study on cyanobacterial lichen (with *Nostoc* as photobiont) in which the photosynthetic activity (CO_2_ uptake or O_2_ evolution on area basis) was found to be appreciably lower in blue light (*λ*_max_ = 463 nm) compared with red light (*λ*_max_ = 637 nm) at irradiances from 20 to 600 μmol photons m^−2^ s^−1^. But the combination of, for example, 25 μmol photons m^−2^ s^−1^ of blue and 25 μmol photons m^−2^ s^−1^ of red light (or 50 μmol photons m^−2^ s^−1^ of red) resulted in much higher rate of photosynthesis than the sum from separate lights, whereas 50 μmol photons m^−2^ s^−1^ of blue light did not produce such effect as red light alone (Solhaug et al. 2014).

The difficulties arise, however, when the content of the individual pigment is dependent not only on light quality during cultivation but also on the duration of the measurement. López-Figueroa et al. (1989) showed in red macroalga *Corallina elongata* that even short-term (30 min) illumination with red light (*λ*_max_ = 630 nm) greatly enhanced the synthesis of Chl *a*, PC, and APC, but not PE. In addition, Ojit et al. (2015) reported that the production of APC in cyanobacterium *Anabaena corcinalis* cultured under red light was three times higher than under blue light. Moreover, the amount of APC may depend on other phycobiliprotein pigments as well. For example, in a strain of cyanobacterium *Synechococcus* (cultured in white light) which, like *C. merolae*, contained only PC, APC constituted as high as 40% (by weight) of the total phycobiliproteins, whereas a strain with PE (50%) and PC (40%) contained only 10% of APC (Lemasson et al. 1973). The unicellular red alga *Rhodella violacea* (cultured in white light), which contained about 60% (by weight) of PE and 25% of PC, had only about 15% of APC (Koller et al. 1977). Thus, it is obvious that the presence of a greater amount of the absorbed red photons (i.e., Chl *a* or APC or both) than blue photons is responsible for higher photosynthetic activity in red light than in blue light. Besides, the amount of blue photons can be reduced by absorption by carotenoids, mainly Zea and β-Car (Takaichi 2011), as well as by other cellular compounds such as flavin nucleotides, flavoproteins, and cytochromes (Lehninger 1976). Red algae, unlike cyanobacteria, do not contain the orange carotenoid protein, which is a blue light-photoactive protein that mediates the thermal dissipation of the excess energy absorbed at the level of PBS (Kirilovsky and Kerfeld 2013).

Due to the fact that the light reactions (O_2_ release) and dark reactions (CO_2_ uptake) of photosynthesis are tightly coupled, the Rubisco enzyme plays a pivotal role in this relationship, especially in the case of thermophilic red alga living in acidic hot springs where the concentration of CO_2_ dissolved in water is rather low (far below 10 μM usually). Nonetheless, the enzyme favors the carboxylase reaction over oxygenase, which indeed takes place in *C. merolae* (Uemura et al. 1997). The effect of light quality on the content/activity of Rubisco in *C. merolae* has not been examined; however, reports are available on this topic in the literature. For instance, Mercado et al. (2002) found that the multicellular red alga *Gracilaria tenuistipitata* cultivated in blue or white light did not differ in the Rubisco content expressed on soluble proteins, although photosynthetic O_2_ evolution in white light (based on cellular area) and chlorophyll content (based on fresh weight) in the thallus grown in blue light was reduced by about 50% and 30%, respectively, compared with the thallus grown in white light. In fern gametophytes grown in blue light, the activity of Rubisco carboxylase was found to be up to threefold higher (per unit enzyme protein) compared to those grown in red light (Eilenberg et al. 1991). It was reported (Eskins et al. 1991) that the activity and amount of Rubisco (based on leaf area) in higher plant soybean (*Glycine max*) were affected by blue and red light just like for fern gametophytes.

In this paper, we describe the findings of experiments with *C. merolae* cells which show that the photosynthetic activity depended not only on the light quality during growth but also on its quality during photosynthesis. The importance of these findings is discussed in relation to chlorophyll and other pigments contents. The involvement of respiration in photosynthesis is also taken into consideration.

## Materials and methods

### Cell culturing and harvesting

The strain *C. merolae* 10D (NIES-1332) was grown in 250 ml Greiner flasks containing 50 ml of Allen 2 medium adjusted to pH 2.5 (Minoda et al. 2004). The alga was cultivated under continuous white light (40 μmol photons m^−2^ s^−1^ from fluorescent tubes LF-40 W, Pila, Poland) at 40 °C, and the medium was aerated by shaking at 120 rpm. When the culture reached a concentration of about 4 × 10^8^ cells/ml (counting with Neubauer chamber), the cells were inoculated into 250 ml fresh medium in 750 ml Erlenmeyer flasks at a density of 5 × 10^5^ cells/ml and were further cultured under the same light conditions. After 10 days, the cultures (at ~ 5 × 10^7^ cells/ml) were irradiated for 6 days with continuous blue or red light (40 μmol photons m^−2^ s^−1^) using panels of light-emitting diodes (LED) with a peak at 460 and 665 nm, respectively. The white light (40 μmol photons m^−2^ s^−1^) was provided by a fluorescent lamp (23 W, OSRAM DULUX EL, Germany). The cultures were vigorously stirred to allow aeration (150 rpm). The cells were then synchronized by subjecting to a 12-h light/12-h dark cycle (Kobayashi et al. 2010). At the end of the second light period, a sample of 10 or 100 ml of cell suspension from each culture was placed in darkness for 18–20 h at 25 °C without stirring to enable cell sedimentation. The harvested cells were washed in Allen 2 medium and centrifuged at 0.6×*g* for 10 min at 25 °C (room temperature). Rademacher et al. (2016) measured the rate of photosynthesis (at 28 °C) also in the cells obtained by centrifugation. The cell pellet was suspended in a small amount of Allen 2 medium (pH 2.5) and stored in the dark for 25–30 min at room temperature before it was used for the determination of photosynthesis and respiration. The preparation of cells for CO_2_ measurements is proceeded as described above and is resulted in obtaining the cells with high photosynthetic activity, compared to that cells collected directly from the growth medium. For extracting the pigments, the nonsuspended pellets were used immediately.

### Measurement of CO_2_ uptake during photosynthesis

Samples of cells (harvested from 100 ml culture) were suspended at a density of ~ 5 × 10^7^ cells/ml in 30 ml of Allen 2 medium (pH 2.5) in a glass-flask photosynthetic chamber (102 ml volume, 30 mm diameter) which was equipped with an inlet with a pored bulbous ending immersed into the suspension and an outlet at the top of the chamber. A magnetic stirrer with a heat regulator was used for maintaining constant temperature (30 ± 2 °C) and mixing (300 rpm). The cells of *C. merolae* were grown at 40 °C whereas CO_2_ uptake (as well as O_2_ evolution) was measured in 30 °C, because it was impossible to measure CO_2_ uptake using CO_2_ analyser in 40 °C due to increased H_2_O interference with CO_2_ in the measuring cuvette. Therefore, the same temperature, i.e., 30 °C, for measurement of photosynthetic O_2_ evolution had to be applied. Some pilot experiment with oxygen electrode show that in the range 30–40 °C, photosynthesis of the investigated cells increased only very slightly.

The chamber was connected to an infrared CO_2_ analyzer (IRGA, Beckman 865, USA) attached to a closed circuit system filled up with air. The air flow rate measured as an input into the chamber was set at 0.67 l min^–1^, which allowed making 1.6 times volume changes per minute at a total system volume of 415 ml. The blue and red light was supplied by LED lamps (PHILIPS Deco, 1 W, GU10, The Netherlands) with bands between 410–500 and 620–700 nm, respectively, while white light (400–700 nm) was provided by a commercial LED BULB (INQ, E27, 10 W, 2700 K, Poland). To establish the width of the spectral bands, a diffraction grating spectrophotometer (Specol 11, VEB Carl Zeiss, Jena, Germany) was employed. The photon flux density (PFD) inside the chamber was 100 μmol photons m^−2^ s^−1^ for each light quality, which was measured using a Quantitherm Light Meter Thermometer QRT1 (Hansatech Instruments Ltd, England). The rate of net photosynthesis (Pn) was determined from the change in CO_2_ concentration in the range of 480–420 μl l^−1^ (i.e., about 12 μM CO_2_ and 230 μM O_2_). The measurements of CO_2_ uptake (in triplicate, light quality in random) were repeated three times in separate experiments for each light quality.

### Measurement of O_2_ evolution/uptake during photosynthesis/respiration

Oxygen evolution in the light (as net photosynthesis, Pn) and oxygen uptake in the dark (as respiration, R) by cells were measured using a Clark-type oxygen electrode (TriOxmatic 300, WTW G.M.B.H., Weinheim, Germany) in 2 ml volume at the same temperature (30 °C). Our data on isolated PSII (Krupnik et al. 2013) show about 20% increase in O_2_ evolution in the range of temperature 30–40 °C during measurements and in higher temperature there was a substantial decrease in oxygen evolving activity. At 30 °C the rate of photosynthesis was high and this temperature was not stressful for cells in any significant measure. When we preincubated cells in room temperature (25 °C), allowing them to settle to obtain cells of only very good physiological state, those cells had very high activity and chlorophyll content did not change. We investigated the fast acclimation to changes in light quality during measurements Pn and R in 30 °C and observed that *C. merolae* cells exhibits stability of photosynthetic apparatus. Before evaluating the photosynthesis, gaseous nitrogen was bubbled through the Allen 2 medium (pH 2.5) to reduce the level of O_2_ to about 30 μM, followed by which 50 μM NaHCO_3_ and cell sample (harvested from 10 ml culture) were added (~ 5 × 10^7^ cells/ml). The suspension was continuously mixed (100 rpm) to prevent cell sedimentation and the formation of an oxygen gradient in the chamber. After 3–4 min of darkness, the photosynthetic O_2_ evolution was recorded until the evolution ceased. Then, the second (and eventually the third) portion of 50 μM NaHCO_3_ was added until CO_2_ was depleted. Only upon the addition of the second (and third) portion of NaHCO_3_, the total evolved O_2_ was in 1:1 stoichiometry with the added amount of CO_2_ (NaHCO_3_), perhaps because some of the endogenous CO_2_ was still present in the cells. The PFD inside the chamber was 60 μmol photons m^−2^ s^−1^ for each light quality, which was a value lower than that for the above-mentioned CO_2_ uptake as the intensity of red light inside the reaction chamber was limited due to its absorption by the water circulated in the outer jacket of the chamber.

The rate of respiratory O_2_ uptake (dark respiration, R) was measured after the dark period (16–18 h) and light period (9–10 h), but using a denser cell suspension (~ 4 × 10^8^ cells/ml) than that used for the evaluation of photosynthesis. Cells from the light period were collected by centrifugation at 0.1×*g* for 10 min at 25 °C. The rate of net photosynthesis was calculated from the change in O_2_ concentration in the range of 45–70 μM O_2_ and respiration in the range of 225–210 μM. The measurements were repeated three times for photosynthesis and six times for respiration after the dark period, while after the light period, the measurements were repeated four to five times.

### Determination of pigments

The cell samples (harvested from 10 ml culture) were diluted to ~ 2 × 10^8^ cells/ml in 2 ml of Allen 2 medium (pH 2.5) in Eppendorf tubes and were centrifuged at 1000×*g* for 10 min at 25 °C. The obtained pellet was suspended in 2 ml of distilled water and heated at 45 °C for 10 min. After heating, PC was extracted by two successive cycles of freezing at -73 °C, thawing in hot water (45 °C), and shaking to disrupt the cells. The extract was centrifuged at 16,000×*g* for 10 min at 25 °C, and a blue supernatant containing the crude aqueous extract of PC was collected. The absorption spectra were determined in the range of 500–700 nm by a spectrophotometer (UV-1800, Shimadzu, Japan) at room temperature, in which one peak was observed at 624.8 nm corresponding to the absorption maximum of c-PC. The remaining green pellet was vigorously agitated with 80% cold acetone for 10 min, and then, Chl *a* and carotenoids were collected in the supernatant after centrifugation at 16,000×*g* for 10 min at 25 °C. The residual blue pellet was treated with distilled water and vigorously stirred. Although the blue pigment was not released into the water, the suspension showed a peak at 650 nm corresponding to the absorption maximum of APC in the eukaryotic unicellular red alga *P. cruentum* (Gannt and Lipschultz 1974). This absorbance was not considered in the evaluation of APC because the proteins precipitate. The concentration of PC in the supernatant was calculated using the following equation derived from the formula of Bennet and Bogorad (1973): PC (mg ml^−1^) = (*A*_624.8_ − 0.474 (*A*_652_)/5.34). The content of Chl *a* and total carotenoids in the supernatant was estimated as described by Wellburn (1994). The estimation of extracted pigments was repeated six times in separate experiments for each light quality.

### Other determinations

Before experiments with light quality, the CO_2_ uptake during photosynthesis in air (21% O_2_, 0.04% CO_2_) and in ~ 1.5% O_2_, which was balanced by the supply of nitrogen with the desired CO_2_ concentration (Parys et al. 1989), as well as the CO_2_ compensation points (Γs) in white light was recorded. When light (100 μmol photons m^−2^ s^−1^) was switched on, the CO_2_ uptake lowered until the unchanged CO_2_ concentration was reached—a Γ value of either 21% or 1.5% O_2_ was attained. In order to identify the intensity of white light that saturated photosynthesis, irradiation of 10–400 μmol photons m^−2^ s^−1^ was applied. The measurements of Γ and Pn at a normal and low level of O_2_ were repeated two times, whereas the effect of light intensity on the rate of Pn was analyzed three to four times, in separate experiments.

The effect of the carbonic anhydrase (CA) inhibitor, ethoxyzolamide (EZ) (Moroney et al. 1985), at a concentration of 50–150 μM, on photosynthetic O_2_ evolution by *C*. *merolae* cells was examined under white light (100 μmol photons m^−2^ s^−1^) in the presence of 50 μM NaHCO_3_, under conditions as described earlier for oxygen electrode. The inhibitor was added from a 10 mM stock solution dissolved in dimethyl sulfoxide (DMSO). The rate of net photosynthesis was calculated from the change of O_2_ concentration in the range of 140–190 μM O_2_. The measurements were repeated three or four times in separate experiments.

For determining the content of carotenoids, analytical high-pressure liquid chromatography was performed, according to a modified method of Krupnik et al. (2013). The process was carried out with a Shimadzu Prominence System and a PDA detector (Shimadzu, Japan), using a maximum flow rate of 1 ml min^−1^ and a Bionacom 3000 C18 column (Bionacom, UK). The pigments were extracted from the cells with 1 ml ethanol. The volume of cell suspension was not greater than one-fourth of the extraction mixture. The cellular and protein debris was removed by centrifugation for 10 min at 4 °C. The extract was concentrated in a SpeedVac centrifuge at 30 °C until it dried out. Samples (20 μg Chl *a*) were dissolved in 50 μl of acetonitrile:triethylamine (99.9:0.1, v/v) mixture and loaded onto the C18 column previously equilibrated with phase A (acetonitrile:methanol, 6:2, v/v). The pigments were separated with a linear gradient of 0–75% of phase B (ethyl acetate), starting at the 10th minute of the run. The content of each carotenoid species was expressed as a molar ratio of a specific carotenoid to Chl *a*, while the concentration was calculated as the area under the pigment-corresponding peak. The molar attenuation coefficients at the wavelength *λ* = 436 nm in acetonitrile were as follows: 91.7, 133.3, and 134.6 mM^−1^ cm^−1^ for Chl *a*, Zea, and β-Car, respectively (Davies 1976). The content of Zea and β-Car was recalculated in relation to Chl *a* in intact cells, and the results were expressed as cell number. The determinations were repeated two times for separate samples.

With the exception of the results shown in Table 1, all the remaining results are expressed in terms of cell number as mean values ± standard errors. The statistical significance of mean differences, if necessary, was determined by one-way ANOVA at the significance level of *p* = 0.05.

**Table 1:** The effect of ethoxyzolamide (EZ) and dimethyl sulfoxide (DMSO) on oxygen evolution of *C. merolae* cells

Treatment		Oxygen evolution (µmol mg^−1^ Chl *a* h^−1^)
EZ (µM)	DMSO (%)	
0	0	83.2 ± 3.2
50	0.5	89.4 ± 3.4
75	0.75	63.9 ± 1.7
100	1	37.6 ± 1.6
150	1.5	35.1 ± 3.7
0	1.5	35.6 ± 4.0

## Results

### Characteristics of photosynthesis in white light

**Fig. 1:**
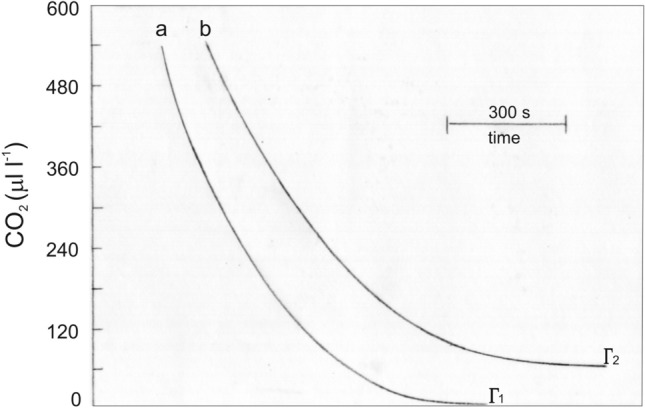
Typical recorder tracings showing the changes of CO_2_ concentration in an atmosphere of 1.5% O_2_ (**a**) and 21% O_2_ (**b**) during illumination of *C. merolae* cells with 100 μmol photons m^−2^ s^−1^ of white light; Γ_1_ and Γ_2_ are the CO_2_ compensation points at low and normal oxygen concentration, respectively. Overlapping charts resulted from shifting back the recorder paper after tracing

**Fig. 2:**
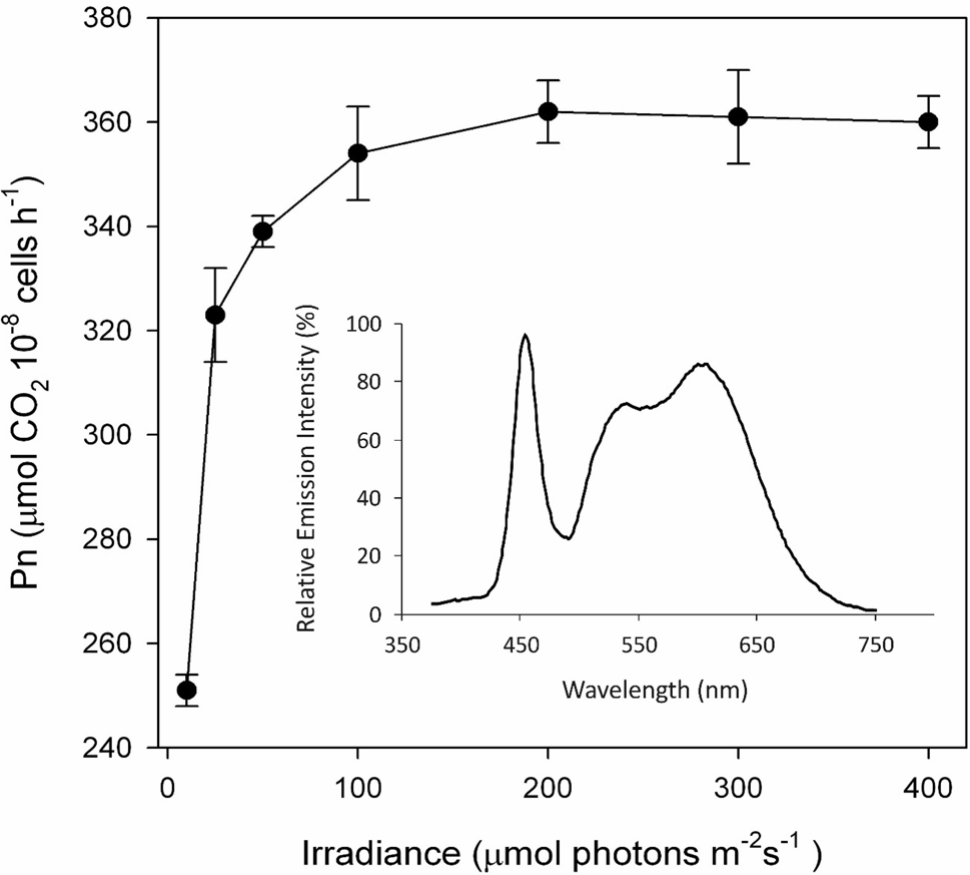
The effect of white light intensity on the rate of net photosynthesis of *C. merolae* cells measured at about 450 μl CO_2_ l^−1^ and 21% O_2_. The insert shows relative spectral distribution of white LED light used for measurements of photosynthesis

The CO_2_ uptake of *C. merolae* during photosynthesis (Pn) revealed that the pattern of CO_2_ fixation by the cells suspended in Allen medium and illuminated with white light (100 μmol photons m^−2^ s^−1^) was the same as that by the leaves of C3 plants in the atmosphere of air O_2_ (21% O_2_) or low O_2_ (i.e., 1–2%). The CO_2_ concentration at CO_2_ compensation point (Γ) in 21% O_2_ reached the value of 60 μl^−1^ (ppm), whereas in low O_2_ (~ 1.5%) concentration, it was close to zero (Fig. 1). The CO_2_ uptake by cells in low O_2_ proceeded at a higher rate (by about 30%) than in the air. On the other hand, experiments with various irradiances revealed that irradiance of 100 μmol photons m^−2^ s^−1^ saturated (up to 98%) the rate of net photosynthesis in air, while the higher irradiance (200–400 μmol photons m^−2^ s^−1^) had no effect (Fig. 2). EZ given at a concentration of 50 μM did not affect the rate of photosynthetic O_2_ evolution, but at a higher concentration (100 or 150 μM), it caused a more than twofold reduction in the photosynthetic activity. However, it seems that the decrease in photosynthesis resulted from the effect of DMSO and not from the effect of EZ (Table 1). Because ethoxyloamide, an inhibitor of intracellular carbonic anhydrase (CA), had no effect on photosynthetic O_2_ evolution when supplied to the cells at 50 μM, our result supports the view that CCM is absent in this alga.

### Effect of light quality on photosynthesis

Our experiments with different colors of light showed that the rate of CO_2_ uptake (Pn) by *C. merolae* was influenced by light quality not only during the cultivation but also during the measurements. The cells grown in blue and red light assimilated CO_2_ at a significantly lower rate (by about 30% and 15%, respectively) than those grown in white light (Table 2). Under blue light, the rate of Pn observed in both white light- and red light-grown cells was twofold lower compared with white control. The other combinations between the light quality during growth and the light quality during photosynthesis measurement showed no significant effect on the rate of CO_2_ fixation, although the rate of photosynthesis in white or red light was sometimes found to be lower by about 20% in comparison with the control value. The dependence of light quality on photosynthetic O_2_ evolution was generally similar to that noted for CO_2_ uptake. Only the cells cultivated in blue light showed a significant reduction (by about 30%) in the rate of photosynthesis (Table 3). Furthermore, when cells grown in white and red light were illuminated with blue light during growth, the photosynthesis rate was reduced to 55% of the control value. The remaining combinations between light quality during growth and photosynthesis did not show any significant difference in O_2_ evolution rates, in spite of lower (by about 20%) rate of Pn in red light for blue light-grown cells. The higher values of O_2_ evolution (50% average) than CO_2_ uptake resulted undoubtedly from the different concentrations of CO_2_ and O_2_ (about 50 and 60 μM, respectively) in liquid medium with oxygen electrode compared to the IRGA system, where the concentration of CO_2_ and O_2_ was about 12 and 230 μM, respectively. If the results are expressed in terms of Chl *a*, then the rate of photosynthetic O_2_ evolution was observed to be almost the same as noticed by Zenvirth et al. (1985) at 300 μmol photons m^−2^ s^−1^ and 30 °C.

**Table 2:** The effect of light quality on the rate of net photosynthetic CO_2_ uptake of *C. merolae* cells

Light during growth	Photosynthesis (nmol CO_2_ 10^–8^ cells h^−1^)		
	White	Blue	Red
White	**366 ± 19**	188 ± 9*	349 ± 15
Blue	291 ± 10	247 ± 18*	301 ± 12
Red	296 ± 19	186 ± 7*	**303 ± 24**

The data represent the mean values ± SE of three independent experiments

Photosynthesis rates at the same light quality as during growth are in bold

Values with asterisk (*) indicate significant differences (*p* = 0.05)

**Table 3:** The effect of light quality on the rate of net photosynthetic O_2_ evolution of *C. merolae* cells

Light during growth	Photosynthesis (nmol O_2_ 10^–8^ cells h^−1^)		
	White	Blue	Red
White	**542 ± 15**	296 ± 20*	489 ± 29
Blue	548 ± 19	389 ± 40*	445 ± 30*
Red	535 ± 15	300 ± 22*	**506 ± 17**

The data represent the mean values ± SE of three independent experiments

Photosynthesis rates at the same light quality as during growth are in bold

Values with asterisk (*) indicate significant differences (*p* = 0.05)

### Effect of light quality on respiration

During dark respiration (R), the rate of O_2_ uptake of *C. merolae* cells was more affected by the period of illumination than by the quality of light maintained during growth. In all the examined cultures, the rate of respiration following illumination was markedly higher (2.5 times on average) compared with the cultures adapted to the dark (Table 4). Unlike photosynthesis, the respiration of cells which were previously grown under blue light and then kept either in the dark for 18–20 h or taken immediately from blue light was significantly higher (by about 30% and 20%, respectively) compared with the cells grown in white light, which might be the reason for the increased synthesis of pigments (Chl *a* and PC) in these cells.

**Table 4:** The effect of light quality on the rate of dark respiration of *C. merolae* cells

Light during growth	Respiration (nmol O_2_ 10^–8^ cells h^−1^)	
	After dark	After light
White	8.0 ± 0.8	23.8 ± 1.5
Blue	10.5 ± 0.5*	29.0 ± 5.5*
Red	10.1 ± 0.7	24.5 ± 7.7

The data represent the mean values ± SE. Oxygen uptake after dark period (16–18 h) was measured in six and after light period (9–10 h) in four to five separate experiments for each quality of light. Values with asterisk (*) indicate significant differences (*p* = 0.05)

### Effect of light quality on pigment contents

It was found that *C. merolae* cells grown in blue and red light contained more Chl *a* (by 25% and 50%, respectively) compared with that grown in white light. On the other hand, the level of total carotenoids increased by 20% only under red light (Table 5), due to an increase in the amounts of Zea (by about 30%) and β-Car (by about 80%) (Table 6). As shown in Table 5, the largest (by 75%) increase in phycocyanobilin (PCB), which is the tetrapyrrole chromophore of PC, was observed under blue light, whereas under red light the increase was smaller (by about 40%). It suggests that *C. merolae* adjust its metabolism to gain an adaptative response to light conditions unfavorable for photosynthesis in blue light. It can also be seen from Table 6 that independent of the light quality during growth the content of PCB chromophores was far less (30% average) than Chl *a* in the algal cells, while the level of total carotenoids was 20% lower but only in the blue light- and red light-grown cells compared with Chl *a*.

**Table 5:** The effect of light quality on chlorophyll *a* (Chl *a*), carotenoids (Car_s_), phycocyanin (PC), and phycocyanobilin (PCB) contents in *C. merolae* cells

Light during growth	Pigment (nmol 10^–8^ cells)			
	Chl *a*	Car_s_	PC	PCB
White	3.47 ± 0.35	3.48 ± 0.56	0.72 ± 0.08	2.16 ± 0,24
Blue	4.33 ± 0.45*	3.34 ± 0.39*	1.25 ± 0.11*	3.76 ± 0.34
Red	5.26 ± 0.55*	4.18 ± 0.49*	1.00 ± 0.14*	3.01 ± 0.41

The content of pigments has been converted from weight to molar quantity according to molecular weight (MW) of 894 Da for chlorophyll *a* (Chl *a*) and 569 Da for carotenoids (Car_s_, zeaxanthin MW was taken) as identified in *C. merolae* (Cunningham et al. 2007). The molecular weight of monomeric form of phycocyanin (PC, 38 kDa) is taken for *Galdieria sulphuraria* (Sørensen et al. 2013), where three phycocyanobilin (PCB) chromophore are attached (Xie et al. 1992). The data represent the mean values ± SE of six separate experiments. Values in each column with asterisk (*) are significantly different at *p* = 0.05

**Table 6:** The zeaxanthin and β-carotene content in *C. merolae* cells growing in white, blue, and red light

Light during growth	Carotenoid pigments (nmol 10^–8^ cells)	
	Zeaxanthin	β-carotene
White	2.18 ± 0.44	0.75 ± 0.07
Blue	2.89 ± 0.33	0.84 ± 0.08
Red	3.34 ± 0.36	1.34 ± 0.19

## Discussion

### Photosynthesis in white light

We found that *C. merolae* had the same pattern of CO_2_ exchange as plants with C3-type carbon metabolism. The CO_2_ compensation point (Γ) of cell suspension saturated with either air (21%) or low (1.5%) O_2_ concentration reached a value of 60 μ l^−1^ or was close to zero, respectively (Fig. 1). It was almost the same as that determined for the leaves of the C3 plant *Festuca arundinacea*, whereas the rate of photosynthetic CO_2_ uptake (Pn) in low O_2_ concentration was higher (by about 30%) than the rate in air (Parys 1990; Parys and Romanowska 2000). Such an effect of oxygen on the Γ and Pn confirms the opinion that photorespiration exists in *C. merolae* cells (Rademacher et al. 2016). We also noticed that ethoxyloamide (EZ), an inhibitor of intracellular CA (Moroney et al. 1985), had no effect on photosynthetic O_2_ evolution when supplied to the cells at 50 μM (Table 1). Since except for mitochondrial CA (γCA class; Gawryluk and Gray 2010) *C. merolae* cells do not possess either chloroplastic or cytosolic CAs (αCA and βCA classes; Meyer and Griffiths 2013), which are necessary for the CO_2_-concentrating mechanism (CCM), our result supports the view that CCM is absent in this alga (Zenvirth et al. 1985; Meyer and Griffiths 2013). Although the αCA enzyme was recently identified in *C. merolae*, its role in supplying CO_2_ to the chloroplasts is not clearly established (Rademacher et al. 2017).

Further examination showed that the cells that were growing in white light (40 μmol photons m^−2^ s^−1^) required around 100 μmol photons m^−2^ s^−1^ of white light only to saturate CO_2_ during photosynthesis (Fig. 2), similarly to Nikolova et al. (2017) who determined O_2_ evolution from *C. merolae* cells cultivated at 70 μmol photons m^−2^ s^−1^. They found changes in remodeling of photosynthetic apparatus induced by temperature, but cells were grown in 25 and 42 °C; thus, they observed stress-related proteins. Also rate of photosynthesis was much lower than in our experiments, but they show that *C. merolae* exhibits stability of photosynthetic apparatus over a great temperature spectrum. Temperature over 40 °C diminished photosynthesis significantly, because the activity of Rubisco activase might be greatly reduced (Yamori and von Caemmerer 2009).

Then, the question arises, however, of why the rate of photosynthesis did not increase with irradiance above 100 μmol photons m^−2^ s^−1^ like it is for plant leaves (Walker 1992). One of the explanations for this is that it resulted from the acclimation of photosynthetic apparatus to light intensity during growth as noted for the unicellular red alga *P. cruentum* cultivated at different irradiances (Cunningham et al. 1992). This view may also support another result which showed that O_2_ evolution from the unicellular green alga *Ostreococcus* strain adapted to 10 μmol photons m^−2^ s^−1^ of blue light was saturated at about 100 μmol photons m^−2^ s^−1^, whereas the strain adapted to 100 μmol photons m^−2^ s^−1^ of white light required up to about 250 μmol photons m^−2^ s^−1^ (Cardol et al. 2008). However, if both strains differed in photosynthesis by twofold at saturation, the latter strain showed only a 10% lower rate of O_2_ evolution at 100 μmol photons m^−2^ s^−1^ compared with the rate at 250 μmol photons m^−2^ s^−1^. Thus, the relation between the saturation of photosynthesis and light intensity during growth may not be as obvious as it seemed. Such an assumption that the amount of CO_2_ in the medium with cells could limit photosynthesis seems unlikely since O_2_ evolution at 50 μM CO_2_ and 60 μmol photons m^−2^ s^−1^ (Table 3) was practically the same as that recorded by Zenvirth et al. (1985) at 50 and 100 μM CO_2_ and about 300 μmol photons m^−2^ s^−1^ of white light. Therefore, our finding may justify why 100 μmol photons m^−2^ s^−1^ of white light is considered in practice as the upper limit at which the cells of *C. merolae* (and other algal species) are cultivated in continuous illumination, as the light above this limit can be deleterious to cell growth due to stress and chlorophyll breakdown (Minoda et al. 2004). The fact that a very low white light (10 μmol photons m^−2^ s^−1^) saturated the CO_2_ uptake up to 70% (see Fig. 2) may suggest that the close relationship between the initial rate of photosynthesis and its saturation point exists.

The blue and red light during the growth of *C. merolae* had little effect on the rate of CO_2_ uptake during photosynthesis in white light, while photosynthetic O_2_ evolution was completely independent (Table 2 and 3). The latter effect was the same as reported for *P*. *umbilicalis*, other red alga species (Figueroa et al. 1995). It is worth to note here that both our results (Table 5) and in the above report indicate that greater amount of pigments absorbing blue and red photons did not lead to a higher rate of photosynthesis in white light. Because our determinations proceeded either in photorespiratory (for CO_2_ fixation) or in non-photorespiratory (for O_2_ evolution) conditions, the independence of photosynthetic O_2_ evolution by blue and red light-grown cells could arise from reduced activity of photorespiration, respiration, or both processes.

As the influence of white light on photorespiration of both blue and red light-grown cells is unknown, it may be assumed that the decrease in photosynthetic CO_2_ fixation by these cells resulted from the mitochondrial respiration (Table 4). Furthermore, a generally accepted theory is that photosynthesis requires appropriate mitochondrial activity (Krömer 1995; Padmasree and Raghavendra 1999); however, the direct determination of respiratory O_2_ uptake or CO_2_ release during photosynthesis is a complicated task (Nunes-Nesi et al. 2007). On the other hand, it is known that photosynthesis results in a temporary increase in the rate of O_2_ consumption (or CO_2_ evolution) in the dark period following illumination. This phenomenon is called light-enhanced dark respiration (LEDR) and is often considered as the indicator of the respiration rate in the preceding light period (Xue et al. 1996). It results presumably from the oxidation of malate and glycine formed during photosynthesis and photorespiration (Parys et al. 2004), but these metabolites were not examined in *C. merolae* cells grown in various light qualities. Instead, the respiration of cells in darkness, before and after a period of illumination during growth, was determined. It appeared that respiration even after illumination would have little (less than 5% average) contribution to the rate of photosynthesis, whereas Nikolova et al. (2017) noticed that O_2_ consumption after photosynthesis in white light by darkened *C. merolae* cells occurred at about 20% of the rate of photosynthesis. This discrepancy may be due to the fact that our cells taken from light were in dark for more than 10 min, and thus, the respiration during LEDR could have been significantly lowered during this period (Reddy et al. 1991; Parys et al. 2004). Nonetheless, *C. merolae* grown in red and blue light showed increased respiration (by about 25 and 30%, respectively) even after a long-term period of darkness (18–20 h) compared with those grown in white light (Table 4), which could be related to increased synthesis of pigments (Table 5). It was shown that both blue and red light are crucial to orchestrate the transcription of gens involved in carbon metabolism and pigment biosynthesis, as blue and red light regulate 35% of the total gens in *C. merolae* (Tardu et al. 2016).

### Photosynthesis in blue and red light

The effect of blue light on photosynthesis of *C. merolae* was the most striking because the same amount of blue as white photons (100 μmol photons m^−2^ s^−1^) resulted in about twofold decrease in CO_2_ uptake or O_2_ evolution by cells that were grown previously in white or red light, but the cells grown in blue light responded much less. The red light did not cause such a drastic reduction of photosynthesis of these cells (by about 10% only), while in those growing in the blue light, it was slightly (by about 20%) greater (Table 2 and 3). So it is clear that the higher photosynthetic activity in red light, compared with blue light, might result from the greater amounts of pigments absorbing red photons. Indeed, cells grown in red light had more Chl *a* (Table 5) and the amount of PCBs, i.e., chromophores of APC, could also be significant as their synthesis depends on the PC content (Lemasson et al. 1973; López-Figueroa et al. 1989). As for blue light, it might be speculated that the decrease in the rate of photosynthesis was due to its reduced accessibility to Chl *a* because the amount of carotenoids was comparable with the content of Chl *a* (Table 5). Most of them constituted Zea (Table 6) which did not transfer blue light energy to Chl *a*, whereas β-Car could transfer (Goedheer 1969; Takaichi 2011) with an efficiency of 30–70% (Ritz et al. 2000) or even 100% (Zigmantas et al. 2002). In *C. merolae*, Zea is mainly associated with the antenna complex of PSI (i.e., LHCI), while β-Car is linked with the PSI core (Tian et al. 2017). These carotenoids are also present in the PSII complex but in much lower amounts (Krupnik et al. 2013). Thus, the screening of Chl *a* by Zea might be responsible for the reduction in the rate of photosynthesis, especially that total amount of Chl *a* and Zea in white light- and red light-grown cells gave the identical Chl *a*/Zea ratio (1.59 and 1.57, respectively), while in cells from blue light, this value was much higher (1.89). It is noteworthy that these ratios well coincide with the photosynthetic activity under blue light; however, this does not explain the mechanism behind it. Due to the fact that our data in this field are insufficient for further discussion, evidence from other studies is used to better clarify this problem. For instance, experiments with cyanobacterium *Synechocystis* sp. strain PCC 6803 suggest that the main reason for the effects of blue light was an excitation imbalance between the photosystems, PSII and PSI, resulting from the low content of Chl *a* in PSII compared with PSI (Luimstra et al. 2018). In *C. merolae*, PSI-LHCI contains about 140 Chl *a* molecules (Tian et al. 2017), while PSII, which exists as a dimmer, contains about 35 Chl *a* molecules on the monomer (Kargul et al. 2012; Krupnik et al. 2013). These numbers are similar to cyanobacteria *Synechococcus* 6301 (Melis 1989) and *Synechocystis* sp. strain PCC 6803 (Luimstra et al. 2018), both of which possess the same set of pigments as *C. merolae*. Besides, cyanobacteria and red algae grown in white light usually have a PSII/PSI ratio of 0.3–0.7 (Melis 1989), though for *Synechococcus* 6301 and unicellular red alga *P. cruentum* a ratio of about 0.5 has often been recorded (Kawamura et al. 1979; Manodori et al. 1984; Cunningham et al. 1989). It may also matter that the overall absorption of light by the PSII-binding PBS in *Synechococcus* 6301 was equal to that of the PSI complex, which suggests that electron flow between the photosystems and energy utilization by the two photoreactions in cyanobacteria and red algae may be balanced despite the low (below 1.0) PSII/PSI stoichiometry (Melis 1989). In our study, the amount of PCB (i.e., chromophore of PC together with APC (not determined)) might be comparable with the overall amount of Chl *a* in both photosystems (Table 5). If it is accepted that most of the Chl *a* was associated with PSI, then it may be assumed that the Chl *a*/Zea ratio in *C. merolae* was mainly referred to PSI-LHCI. Photosynthesis proceeded at a twofold higher rate in saturated white light, compared with blue light, because blue photons absorbed by overall Chl *a* provided only half of the energy utilized by the two photoreactions. Moreover, with Chl *a* screened by Zea (and partly by β-Car), the amount of blue photons absorbed by PSII might be the same as that absorbed by PSI, which balanced the electron flow and energy utilization by the two photoreactions. In other words, photosynthesis in blue light may depend on the accessibility of blue photons to Chl *a* rather than its overall amount, although the stoichiometry of photosystems formed at a given light quality during growth can also be relevant. It was already mentioned that blue light-grown *C*. *merolae* performed more (by about 30%) photosynthesis in blue light compared with that grown in white or red light. In this case, the Chl *a*/Zea ratio was also higher (1.89), whereas the enhanced synthesis of carotenoids in red light-grown cells led to the reduction of Chl *a*/Zea ratio (1.57) to the same value as in the cells grown in white light (1.59). Moreover, PSII in cyanobacteria and red algae could contain from 35 to 60 Chl *a* molecules depending on the light-growth conditions, while in PSI, the level of Chl *a* was not so susceptible to light conditions (Diner and Wollman 1979; Myers et al. 1980; Manodori and Melis 1986). For this reason, cyanobacterium *Synechocystis* sp. strain PCC 6803 acclimated to blue light showed a much higher PSII/PSI ratio (0.83) compared with that acclimated to orange or red light (0.25) (Luimstra et al. 2018). It is thus very likely that *C. merolae* cells grown in blue light had either increased PSII/PSI ratio compared with those grown in white or red light or an increased amount of Chl *a* in PSII, which led to their higher rate of photosynthesis in blue light as noted for CO_2_ uptake or O_2_ evolution. In turn, the low rate of photosynthesis of cells in the blue light which resulted from the use of white or red light during growth might be interpreted by the reduced PSII/PSI ratio.

A possible is also such explanation that blue light absorbed by Chl *a* in the small inner antennas of PSII (CP43 and CP47; Krupnik et al 2013) did not provide enough excitation energy to the RC. It may be because chlorophyll absorbs not more than one photon every 0.1 s, even from intense sunlight, which means that an RC without extensive antennas would remain inactive most of the time (Blankenship 2002). One might speculate that a twofold fall in the rate of photosynthesis in blue light in the case of *C. merolae* cells grown in red and white light (in which the Chl *a*/Zea ratio was low) resulted from the twice lower supply of excitation energy to the RCs of both photosystems. The fact that the blue light-grown cells with a higher Chl *a*/Zea ratio showed higher photosynthesis in blue light may confirm the above explanation. Then, the rate of photosynthesis of blue light-grown cells in white light would be expected to be higher than the control rate; however, such an effect was not seen in our study. Nonetheless, it was observed by others (Aguilera et al. 2000) when, unlike our study, they used unsaturated light. There is also another problem related to blue light and photosynthesis. Some brown algae require only 0.5% of blue light for saturating irradiance with yellow or red light in order to increase the rate of photosynthesis to the value as in white light (Dring et al. 1994). This suggests that blue light, in addition to providing energy for photochemistry, can increase the energy transfer from the antennas to the RCs. Recent studies suggest that the efficiency of energy transfer in light-harvesting complexes depends on the molecular vibrations of pigments together with the related proteins that accompany the absorbed light (Caruso et al. 2009; Scholes et al. 2012; Brédas et al. 2017). It is therefore possible that a part of the energy coming from blue light which cannot be used in photosynthesis (i.e., thermal energy) could thereby increase the vibrations during energy transfer.

## Conclusions

Our data showed that the blue and red light influence photosynthesis in *C. merolae* by adjusting its metabolism to unfavorable conditions for photosynthesis of light, inducing changes in pigments content and respiration rate.

Our work seems to raise several new questions on the photosynthesis in variable amounts of blue, red, and yellow-orange light photons, used separately and in combinations. Also gradual production of cells with genetically reduced or inactivated phycobilisomes would be helpful for understanding how this alga can optimize photosynthesis in variable environmental light conditions.

## Data Availability

Data were not deposited in repository center.

## Abbreviations

APC: Allophycocyanin
β-Car: β-Carotene
Γ: CO_2_ compensation point
CA: Carbonic anhydrase
CCM: CO_2_-concentrating mechanism
Chl a: Chlorophyll a
DMSO: Dimethyl sulfoxide
EZ: Ethoxyzolamide
LEDR: Light-enhanced dark respiration
PBSs: Phycobilisomes
PCB: Phycocyanobilin
PE: Phycoerythrin
PFD: Photosynthetic flux density
Pn: Net photosynthesis
R: Dark respiration
Zea: Zeaxanthin
